# Understanding RNA modifications: the promises and technological bottlenecks of the ‘epitranscriptome’

**DOI:** 10.1098/rsob.170077

**Published:** 2017-05-31

**Authors:** Matthias Schaefer, Utkarsh Kapoor, Michael F. Jantsch

**Affiliations:** Center for Anatomy and Cell Biology, Medical University Vienna, Schwarzspanierstrasse 17-I, 1090 Vienna, Austria

**Keywords:** RNA, transcriptome, chemical modification, epigenetics

## Abstract

The discovery of mechanisms that alter genetic information via RNA editing or introducing covalent RNA modifications points towards a complexity in gene expression that challenges long-standing concepts. Understanding the biology of RNA modifications represents one of the next frontiers in molecular biology. To this date, over 130 different RNA modifications have been identified, and improved mass spectrometry approaches are still adding to this list. However, only recently has it been possible to map selected RNA modifications at single-nucleotide resolution, which has created a number of exciting hypotheses about the biological function of RNA modifications, culminating in the proposition of the ‘epitranscriptome’. Here, we review some of the technological advances in this rapidly developing field, identify the conceptual challenges and discuss approaches that are needed to rigorously test the biological function of specific RNA modifications.

## 1. Introduction

**Figure 1.**
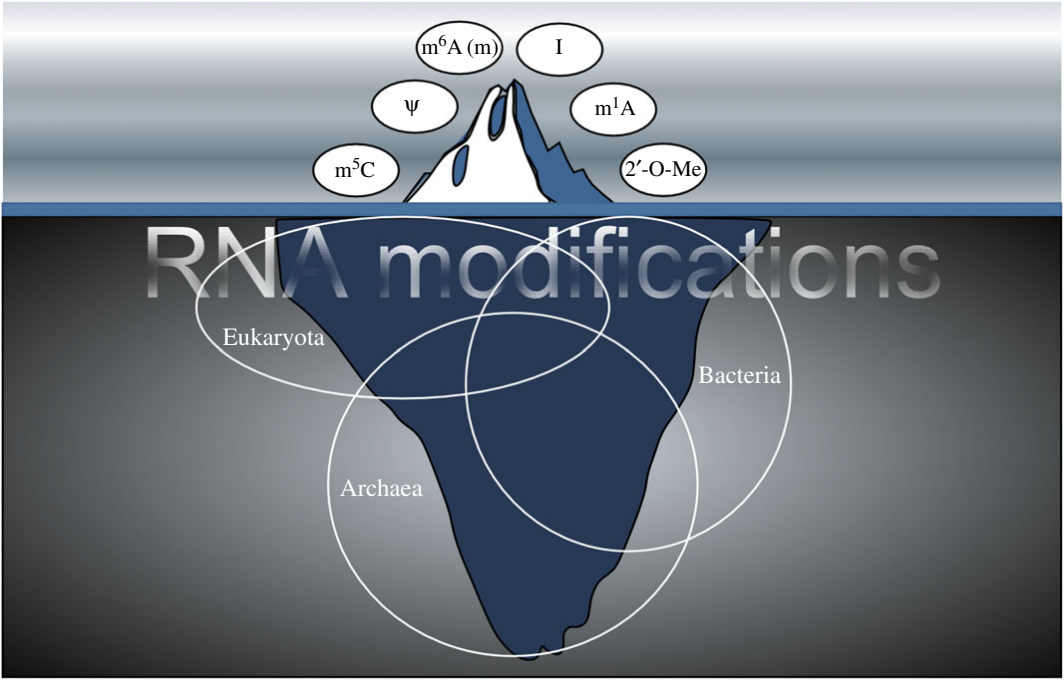
Modifications under the surface of detection. Improved sequencing methods have led to the discovery of millions of modification sites in all classes of RNAs. However, efficient detection of modifications is mostly possible at sites that undergo deamination. Given that more than 130 types of modified ribonucleotides are known to date, it can be expected that novel technologies will lead to a huge increase in detectable RNA modifications that are currently hidden underneath the (detection) surface.

Gene expression is a multi-layered process that starts with controlling access to particular sequence information encoded in DNA, followed by copying this information to RNA molecules, which then branch off into transferring their sequence information into polypeptides (as coding RNAs, cRNAs), or which function as non-coding RNAs (ncRNAs). Importantly, the functionality of RNA does not only rely on sequence information. While splicing, and in particular alternative splicing, can diversify RNAs, each and every RNA nucleoside can be chemically modified or even interchanged (RNA-edited). Presently, over 130 post-synthetic RNA nucleoside modifications can be distinguished in different kingdoms [1]. Although the functions of most of these modifications are largely unknown, their presence in many species point towards evolutionarily conserved molecular toolboxes that may modulate the flow of genetic information or allow reacting to environmental challenges. Deciphering how exactly such modulation is achieved is an exciting new frontier in biology. Most of the known RNA modifications map to abundant RNAs (transfer RNA, tRNA; ribosomal RNA, rRNA). Therefore, our understanding of the molecular function of RNA modifications has mostly been shaped by work on tRNAs and rRNAs. In addition, modifications of eukaryotic cRNA ends (i.e. 5′-capping, 3′-tailing) have long been considered to be the only relevant post-transcriptional changes to mRNA. However, already the discovery of intricate and cell-type-specific RNA splicing patterns and the widespread occurrence of RNA editing events indicated that RNAs do not necessarily function as primary transcripts [2,3]. Moreover, internal cRNA modifications such as methyl-6-adenosine (m^6^A), methyl-5-cytosine (m^5^C), ribose-methylation (2'-O-Me) and pseudo-uridine (Ψ), although known for over 50 years [4–8], have not been accessible to molecular investigation until very recently. Thanks to improved methodology, including next-generation sequencing (NGS) as well as mass spectrometry, an ‘explosion’ of activity in RNA modification research has started a feverish race aiming to comprehensively map specific RNA editing and modification patterns transcriptome-wide and in various tissues and cell types. These are exciting times, not only for RNA biologists, but also for structural and systems biologists. However, it is important to point out that many of the conclusions in this rapidly developing field are still based on manipulating a limited number of experimental model systems with the focus on a few tractable RNA modifications, while the function of the majority of RNA modification has not been addressed (figure 1). And even though thousands to millions of specific RNA modification events (covalent and edited, respectively) have now been mapped to particular sequences, very little or rigorous follow-up experimentation has been conducted. Consequently, while quantitatively more is known about the position of particular RNA modification sites, obtaining functional insight is lagging behind. Despite these shortcomings, already the mapping of inosine, m^6^A, Ψ, m^5^C or m^1^A, especially to low-abundance RNAs (i.e. cRNAs and long ncRNAs), has given rise to testable hypotheses and has opened new avenues for exploration. Many recent reviews described the technical details of current RNA modification mapping approaches and also discussed the seminal studies in the field. Instead of reiterating the content of other reviews, we will only briefly introduce the technological advances for mapping RNA modifications, but will instead focus on conceptual and technical challenges that arise when studying complex biological systems involving only a few catalytic entities affecting a multitude of substrates. Because of the recent introduction of terms such as ‘RNA epigenetics’ and ‘epitranscriptomics’ into the field, we also aim to critically discuss their definition, especially in the light of an invoked similarity to the complexities observed for epigenetic gene regulation systems.

## 2. RNA modification research on the move: recent advances

**Figure 2.**
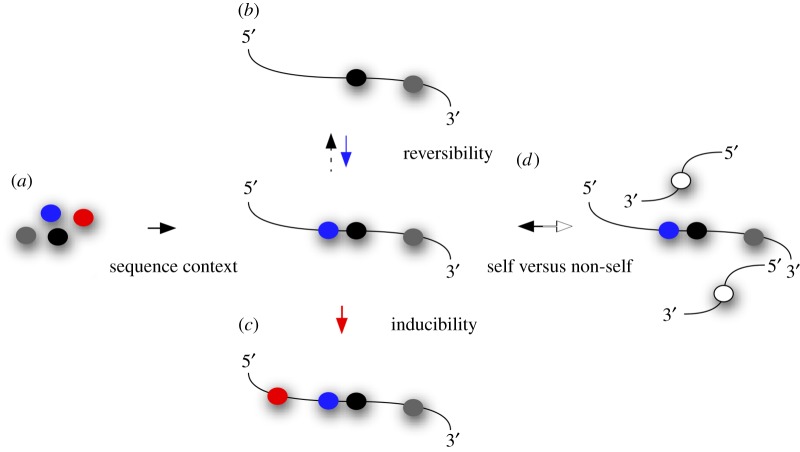
Advances in conceptualizing the dynamic ‘epitranscriptome’. While sequence context-dependent mapping has become a technical reality (*a*), it is currently not clear how RNA modifications influence each other. Some (but not all) modifications have been shown to be reversible (*b*), while other modifications have been shown to be inducible (*c*). The mechanisms of induction and removal of modifications, and in particular the regulatory mechanisms underlying the dynamic landscape of RNA modifications, are poorly understood. As some RNA modifications are specific for certain phyla, their presence or absence can be interpreted as pathogen-associated molecular patterns (PAMPs) and therefore help to distinguish self- from non-self RNAs (*d*).

Understanding the development of organisms has been the focus of most experimental biology during the last 100 years. Ever since covalent RNA modifications were linked to amphibian oocyte development [9], conceptualizing their molecular function centred mostly on cellular proliferation and differentiation. Especially, mutations in tRNA modification enzymes caused developmental aberrations, affected organismal fitness or were incompatible with life altogether (reviewed in [10–13]), which is not surprising, given their prominent roles in protein synthesis. By contrast, no single rRNA modification was found to be essential for ribosome function under standard conditions [14,15]. In support of this notion, ribosomal assembly can be achieved using *in vitro*-transcribed 23S rRNA [16], which even allows for peptidyl transfer [17], but also *in vitro*-transcribed tRNAs lacking all modifications are functional in reconstituted protein translation assays [18]. In addition, some tRNA modification mutant phenotypes can be rescued by overexpressing respective tRNAs [19,20], indicating that post-transcriptional RNA modifications are not essential to the basic function of RNAs (at least in protein translation), pointing towards context-dependent functions. Indeed, the phenomenon of codon bias has placed tRNAs and their modifications into a context-dependent light [21–24]. Four major advances have contributed to conceptualizing biologically relevant functions of RNA modifications (figure 2).

### 2.1. Improved methods for sequence context detection

While physico-chemical detection methods can only report on the presence or absence of a modified nucleoside, adapted NGS technology provided evidence for the notion that virtually every RNA species, including lowly expressed RNAs, is decorated with specific RNA modifications. NGS data mining also pointed to commonalities in the local clustering of particular RNA modifications at functional sequence features, especially in cRNAs such as in untranslated regions (UTRs), transcriptional start sites, exons and introns. For instance, m^6^A was mostly mapped to the last exon in the majority of cRNAs indicating regulation of 3′ UTR function [25]. By contrast, m^1^A was enriched in the 5′ UTR and around start codons of human and mouse mRNAs [26,27], indicating roles that are different from m^6^A. Furthermore, while Ψ appeared to cluster in mRNA coding sequences [28], m^5^C was mapped at 5′ and 3′ UTRs in mRNAs of a highly unstable cancer cell line [29], at translation start sites in mouse embryonic stem cells (mESCs) and whole brain tissues [30], but also to coding sequences in different mouse tissues [31] and in *Arabidopsis* [32]. NGS data also revealed that the majority of A-to-I RNA editing events occur in mobile element-derived sequences [33], and not in cRNAs to which editing events had been assigned earlier [34].

### 2.2. The potential for reversibility

The discovery of enzymatic activities that remove RNA modifications, although presently only documented as integral parts of adenosine methylation systems (reversing m^6^A, m^6^Am and m^1^A) [27,35–37], pointed towards the responsiveness of these modification systems to signals eliciting RNA repair [38] or removal of modified entities when required. In addition, modifications, if not reversed to the unmodified state, can be further modified such as, for instance, Ψ by N1 methylation [39], m^5^C to various oxidation products by the activity of ten-eleven translocation family enzymes [40] or 3-methylcytosine (m^3^C) to 3-methyluridine (m^3^U) [41].

### 2.3. Dynamic regulation

Exposure of cells or organisms to non-laboratory conditions revealed dynamic responses of RNA modification systems to various stresses. For instance, mass spectrometric analyses detected tRNA modification changes upon exposure to mechanistically different toxins [42], which affected codon usage indicating that stress-specific reprogramming of nucleoside modification contributes to translational control [23,43]. Of note, modifications in rRNA and tRNA are especially abundant in thermophilic organisms, suggesting functional roles at elevated temperatures [44]. For instance, RrmJ (FtsJ), a well-conserved heat-shock protein, is highly induced upon heat stress when it catalyses 2′-O-Me at exactly one U in 23S rRNA affecting the (A)1-site of bacterial ribosomes [45]. Interestingly, Cfr, an enzyme generating C8-methyladenosine, targets bacterial 23S rRNA upon environmental insult, causing resistance to several ribosome-targeted antibiotics [46]. Furthermore, heat shock increased m^6^A (or m^6^Am) in 5′ UTRs of mammalian cRNAs, thereby promoting cap-independent translation [47,48]. Also, stress conditions resulting in growth arrest increased m^5^C at specific positions in yeast tRNA [49], and the activity of Pmt1, a (cytosine-5) RNA methyltransferase homologue, was strongly stimulated by the microbiome-dependent tRNA modification queuosine [50]. Similarly, nutrient deprivation in yeast and serum starvation of human cells induced RNA pseudo-uridylation [51,52]. These findings and the identification of RNA editing and modification events in post-mitotic and adult tissues [53–56] bear witness to the notion that RNA modifications are dynamically placed, can be further modified, repaired or even removed in response to events that are not developmentally programmed, but allow organisms to react to changing environments.

### 2.4. Molecular pattern recognition determination

RNA modifications contribute to immune system function by acting as discriminators between RNAs originating from different phyla. For instance, modified nucleosides such as m^5^C, m^6^A, m^5^U, s^2^U or Ψ suppressed signalling of innate RNA sensors such as human toll-like receptors TLR3, TLR7 and TLR8 [57]. Furthermore, a link between MDA5-mediated viral mRNA sensing and 2'-O-Me suggested that RNA modifications act as molecular signatures for the discrimination between RNAs [58]. Supporting this notion, a single 2′-O-Me on G_m_18 in tRNA was sufficient to suppress immune stimulation through human TLR7, indicating that, beyond its primary structural role, 2'-O-Me acts as TLR7 signalling antagonist [59,60]. Of note, one isoform of mammalian ADAR1 (p150) contains an interferon-inducible promoter, p150 shuttles between the nucleus and cytoplasm, and activated p150 in virus-infected cells caused an increase in detectable inosines in cellular RNAs [61]. Furthermore, single-stranded and inosine-containing RNAs, after uptake by scavenger class-A receptors and signalling through TLR3 and dsRNA-activated protein kinase, can stimulate the innate immune system [62]. These findings established that RNA modifications facilitate distinguishing host RNAs (self) from foreign RNAs (non-self) [63,64]. Importantly, the biological effects of specific RNA modifications, when introduced into synthetic RNAs, have contributed to the ‘second coming’ of RNA therapeutics [65]. For instance, replacement of every fourth uridine and cytidine with 2-thiouridine and m^5^C, respectively, decreased binding of synthetic mRNAs to pattern recognition receptors (i.e. TLR3, TLR7, TLR8, RIG-I) in human blood cells [66]. Developing small-interfering RNAs, RNA-based vaccines or mRNA therapeutics regularly includes modifying component RNA strands, which decreases nuclease sensitivity and reduces the activation of the innate immune response [67–70]. Furthering our understanding of the discriminatory potential of specific RNA modifications is holding immense promise for increasing tissue delivery and cellular uptake properties of RNAs, which are still important hurdles to the informed design of RNA-based therapeutics [71]. In summary, recent insights into the placing, dynamics, potential reversibility as well as the immunogenicity of specific RNA modifications are major advances in the field, which are crucial for defining common functional denominators that are needed when conceptualizing the context-dependent roles of RNA modifications.

## 3. RNA modification research comes of age: conceptual challenges

**Figure 3.**
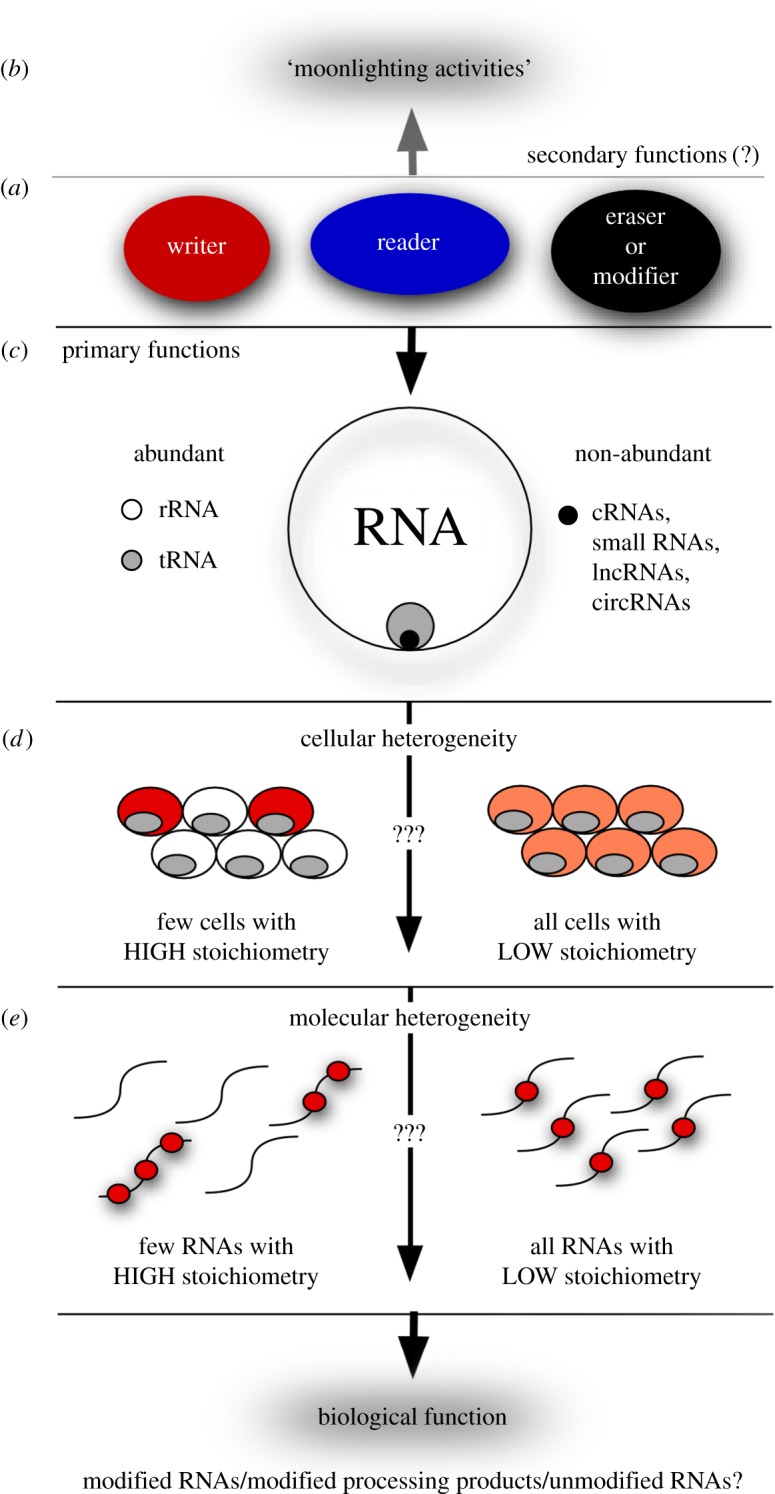
The challenges of the multi-layered world of RNA modifications. RNA modifications are introduced by ‘writer’ enzymes that can then be interpreted by ‘reader’ proteins before being physically removed by ‘erasers’ or further modified by ‘modifiers’ of modifications (*a*). While few modifications have been described that employ all three classes of proteins, it is conceivable that readers and erasers exist for many more modification types than is currently appreciated. Moreover, all classes of proteins may exhibit moonlighting functions (*b*), in addition to their reported modification-related activity. The majority of RNA modifications occur in abundant RNA species, such as rRNAs (greater than 90% of all RNAs) and tRNAs (greater than 5% of all RNAs), but also non-abundant RNAs (less than 5% of all RNAs including cRNA, small RNAs, lncRNAs, circRNAs) carry modifications (*c*). Presently, it is impossible to distinguish whether a given RNA modification in a population of cells is very abundant in some but completely absent from other cells, or alternatively, whether all cells in a population contain a rather low abundance of this particular modification (*d*). Lastly, even within individual cells, it is unclear whether individual RNAs carry multiple marks of the same modification, while other RNAs remain unmodified, or alternatively, whether modifications are distributed rather homogeneously among all transcripts (*e*). Addressing these questions will be the next challenge in RNA modification research.

Understanding the biological impact of modified RNA nucleosides that are often non-abundant and respond dynamically to environmental conditions poses various conceptual challenges (figure 3).

### 3.1. The impact of modifications in high versus low abundance RNAs

The majority of documented RNA modifications map to abundant ncRNAs (rRNA, and tRNA) [72]. For instance, up to 25% of all nucleotides in tRNAs can be modified. Measured RNA modification stoichiometry in ncRNAs indicates biological relevance [73]. About 1% cellular RNA has coding potential and only a fraction of these cRNAs are modified. In addition, other low-abundance RNAs such as small RNAs and long ncRNAs (lncRNAs) carry modifications. Even circular RNAs (circRNAs) are m^6^A-modified [74], and high expression of circular RNAs in the nervous system, robust A-to-I editing in brain tissues and ADAR1 knockdown-induced circRNA expression point towards a role for RNA editing in circRNA function [75]. Of note, only a fraction of these low-abundance RNA species appears to be modified by the same enzymatic machineries that also target abundant ncRNAs. This poses the challenge of how to separate the biological effects of an RNA modification at a modifiable position in abundant versus low-abundance RNA.

### 3.2. Biological function of modified RNA turnover products

Secondary products originating from mature RNAs that were considered to be metabolically stable might also be functionally relevant. For instance, stress-induced processing of various small RNAs (i.e. tRNAs, snoRNAs, vaultRNAs) into smaller RNAs has been observed [76–78]. Whether or not the modification status of these secondary RNAs is different from that of their parental molecules and contributes to their potential functions remains to be tested [79]. The challenge will be separating the functional impact of RNA modifications on molecules of origin and their processing products.

### 3.3. Appreciating the heterogeneity of RNA modifications

The high stoichiometry of some tractable RNA modifications, especially in ncRNAs, gave rise to the notion that RNA modifications are either present or absent from an RNA species. However, this binary view was challenged by findings showing that tRNA isoacceptors can be incompletely modified at specific positions [80–82]. Recent NGS-based mapping data also indicated the substoichiometric occurrence of RNA modifications in cRNAs. Is the seemingly low occurrence of RNA modifications at sequence-identical RNAs a result of analysing heterogeneous cell populations, failure to exclude technical and analytical artefacts, or does it reflect reality? These questions underscore that accurate quantification of modified nucleosides at high sensitivity remains a pressing technical problem. However, if such substoichiometry reflects cellular reality, how can one conceptualize the biological impact of an RNA modification that only occurs on a few of many sequence-identical RNAs?

### 3.4. Approaching the single cell ‘epitranscriptome’

Presently, the detection and functional analysis of RNA modifications uses and addresses entire cell populations. Given the low stoichiometry of cRNA modifications, key to assigning biological function will be quantifying actual copy numbers of individual RNAs that are modified, preferably in single cells. Single molecule detection using advanced hybridization and imaging techniques revealed that, on average, mammalian cells only contain 50–70 copies of an individual and well-expressed mRNA, while some mRNAs (i.e. coding for transcription factors) are only present in single-digit copy numbers [83,84]. Furthermore, single-cell RNA sequencing approaches revealed not only quantitative expression differences between cell types [85] but also expression fluctuations in cell populations of the same genetic origin and subtype [86,87]. Therefore, a combination of quantifying RNA copy number and RNA modification status is needed to arrive at new concepts that help distinguishing biologically meaningful signals from background noise.

### 3.5. Understanding loss-of-function phenotypes

Deducing biological function often relies on interpreting the effects of gene knockouts or targeted mutations reducing catalytic activity of enzymes. In the case of RNA-modifying enzymes with multiple substrates, it is unclear how to assign mutant phenotypes to loss of modification in specific RNA species. For instance, the NOP/Sun2RNA methyltransferase family member 2 (NSUN2) targets the majority of nuclear-encoded tRNAs, but also other cRNAs and ncRNAs [78,88]. A genetic knock-out in mice caused a variety of phenotypes ranging from impaired cellular differentiation to sex-specific infertility [89,90], while human individuals with congenital mutations in a splice-acceptor site of the NSUN2 gene display pleiotropic phenotypes defined by intellectual disability and skin differentiation defects [91,92]. Which methylation site(s) in which RNA substrate(s) cause(s) these phenotypes? While NSUN2 is a robust tRNA methyltransferase on (many) tRNAs, it is unclear which non-modified tRNA species or if any other RNA substrate contributes to the observed pleiotropy of mutant phenotypes. Also, loss of inosines from the transcriptome results in pleiotropic effects. In mammals, adenosines are deaminated to inosines by ADAR1 or ADAR2 [93]. Inosines are interpreted as guanosines during splicing or translation. Most interestingly, ADAR1 and ADAR2 have partially overlapping and distinct substrate specificities [94–96], while loss-of-function phenotypes of both enzymes differ considerably. ADAR2 deletion causes early post-natal lethality in mice, which can be rescued by genomic replacement of a single A-to-G in the *Gria2* gene mimicking an abundant editing event at the corresponding site in the mRNA [97]. By contrast, ADAR1-deficient mice die during embryogenesis but can be rescued by deletion of viral RNA sensors, indicating that the presence of inosines in cellular RNAs is required to suppress the activation of innate immunity sensors [98–100]. Interestingly, inosines introduced by ADAR2 are either numerically insufficient or are wrongly placed to effectively suppress innate immune signalling. Furthermore, rescue of ADAR2 deficiency by a ‘pre-edited’ *Gria2* allele raises the question as to the functional significance of the many other ADAR2 sites in coding regions, the elucidation of which may require further experimentation beyond the observation of loss-of-function phenotypes under standard laboratory conditions. Similar considerations apply to any multi-substrate RNA modification system, which presents an important conceptual challenge when interpreting RNA modification system mutant phenotypes.

### 3.6. Distinguishing primary and secondary protein functions

Some RNA modification enzyme null mutant phenotypes are only partially recapitulated in mutants harbouring catalytically dead versions, indicating limits to simplified genotype-to-phenotype inference [101,102]. For instance, a full deletion of the yeast m^6^A methyltransferase IME4 displayed more severe phenotypes than those caused by a catalytic mutant, suggesting that IME4 may have RNA methylation-independent functions [103,104]. Similarly, METTL3 promotes translation in human cancer cells but independently of m^6^A catalysis [105]. Null mutations in the human FTO gene, an AlkB subfamily member that reverses m^6^A methylation through oxidation, link to an autosomal-recessive lethal syndrome in humans [106], and individuals with intron mutations in FTO displayed increased risk of obesity and type 2 diabetes [107,108], indicating that m^6^A RNA methylation plays a role in the aetiology of metabolic diseases. However, a recent study showed that single-nucleotide polymorphisms (SNPs) in this FTO intron affect the promoter of IRX3, a transcription factor linked to the regulation of body mass in mice, and not FTO expression [109]. Even ADAR1 may have editing-independent functions because a catalytically dead ADAR1 mutation can be fully rescued by a mutation in the viral RNA sensor MDA5, while the same sensor mutation is unable to fully rescue a complete deletion of ADAR1 [100,110]. Supporting the notion of editing-independent functions for ADAR1, overexpression of a catalytic mutant ADAR1 protein in *ADAR1* null human ESCs rescued neuronal differentiation defects by affecting miRNA processing [111]. This suggests that ADAR1 may be required for other functions beyond adenosine deamination. Indeed, enzymes can gain activities that are unrelated to their canonical functions. Such ‘moonlighting’ activities have been reported for glycolytic enzymes that shuttle into the nucleus (reviewed in [112]) and metabolic enzymes that display context-dependent RNA binding activities (reviewed in [113]), which might even affect the regulation of RNA-modifying enzymes as observed for mitochondrial tRNA methylation [114]. Therefore, a critical challenge is to link RNA modification system mutant phenotypes to a particular nucleotide modification and to separate them from secondary functions of the associated proteins.

### 3.7. Semantics

The term ‘RNA epigenetics’ has been coined by invoking similarities between the reversibility of epigenetic DNA or protein modifications and the dynamic nature of one RNA modification, m^6^A [115]. The potential for modification reversibility is a major part in the epigenetic concept, which poses that functionally relevant changes to the genome brought about by epigenetic mechanisms do not involve nucleotide sequence changes and can be inherited (reviewed in [116]). Accordingly, ‘RNA epigenetics’ would encompass inheritable and functionally relevant RNA modifications, which do not involve nucleotide sequence changes and are reversible. However, such a stringent definition would exclude a number of RNA modifications, especially those that have not been observed to be directly reversible (i.e. Ψ to U, m^5^C to C, and various editing events by, for instance, reamination) unless one considers further modification of chemical groups as a kind of reversal which could account for neutralizing the impact of the original modification-intrinsic feature. In addition, the heritability of modified RNAs (through cell divisions or generations) has only recently been experimentally addressed. While RNA modifications have been detected in sperm-derived RNA [117], a maternally provided m^6^A reader protein was shown to mediate accelerated degradation of maternal RNAs in zebra fish, thereby contributing to maternal–zygotic transition (MZT) [118]. However, experimental proof for reversibility and inheritance-related biological functions of other RNA modification systems needs to be provided before labelling all RNA modifications as functional in an epigenetic sense. A more general term, ‘epitranscriptomics’, was introduced after m^6^A mapping studies showed widespread occurrence in every RNA sequence context [119–121]. Meanwhile, research into the molecular function of adenosine modification systems is fast progressing, implicating m^6^A, m^1^A and m^6^Am in regulating mRNA splicing, export, stability and translation. In fact, the term ‘epitranscriptomics’ is now almost exclusively used for RNA modification systems that target cRNAs [122–125]. This raises the question of whether RNA modifications on abundant ncRNAs should be excluded from the concept of the ‘epitranscriptome’. The recent reporting on the function of tRNA m^1^A demethylation in the control of protein translation is a reminder that this would be a mistake [126]. Furthermore, agreeing on an all-encompassing ‘epitranscriptome’ definition will be a more daunting task than defining the ‘epigenome’, which, by utmost simplification, is present only on one or two copies of nuclear DNA. By contrast, RNA modification systems act on more than two copies of sequence-identical RNAs, and often on different RNA species. Dissecting such complexities on individual RNA modification systems first might reveal common denominators allowing the synthesis of a more widely applicable definition for the ‘epitranscriptome’ in the future.

## 4. Detecting RNA modifications: a prerequisite for addressing function

Determining the biological function of RNA modifications requires methodology that allows distinguishing unmodified from modified nucleosides, mapping their distribution and quantifying modified positions. For many years, RNA modifications could mostly be detected by means of their physico-chemical properties using chromatographic methods and mass spectrometry [127]. While these techniques reported mostly on abundant modifications in equally abundant RNA species (i.e. tRNAs, rRNAs), rare modification events, especially in low-abundance RNAs, went largely unnoticed. The single major technological advance that ‘restarted’ and crucially expanded the scope of the RNA modification field was the application of massive parallel sequencing technologies to the detection of low-abundance RNA modification events. Most importantly, NGS-based technologies either preserve or allow deducing RNA sequence context, a necessary prerequisite for experimentally testing the function of a particular RNA modification in a particular RNA sequence. Because the last 5 years of RNA modification research can be described as one large mapping expedition, we will briefly summarize the three basic approaches to using NGS technology for RNA modification mapping.

### 4.1. Direct RNA modification mapping

Currently, only RNA editing events, which all involve deamination reactions, can be mapped directly because the identity of a deaminated nucleotide can be inferred from its cognate pairing during the reverse transcription reaction, which, when compared with the genomic reference sequence, allows for modification calling. The very first attempt to map RNA modifications on a transcriptome-wide scale made use of sequence information of expressed sequence tags and predicted thousands of A-to-I editing events in humans [128]. Only the introduction of NGS technology allowed more cost-effective experimentation, resulting in crucial insights into the extent of A-to-I editing [129] and revealing that editing is a tissue-specific and developmentally regulated phenomenon [130,131].

### 4.2. Indirect modification mapping without prior enrichment

All indirect RNA modification-mapping methods without prior RNA enrichment rely on the differential reactivity of a canonical nucleoside when compared with its modified nucleoside. Many such methods exploit the fidelity and/or processivity of reverse transcriptases (RT) allowing ‘RNA modification calling’ at sites that interfered with RT [132]. For instance, all detection methods for pseudouridine (Ψ) employ treatment of RNA with *N*-cyclohexyl-*N*′-(2-morpholinoethyl)-carbodiimidemetho-*p*-toluenesulfonate (CMCT), which introduces Ψ-CMC adducts that terminate cDNA synthesis, thereby revealing potential Ψ positions. This selective labelling chemistry has been combined with NGS to develop Ψ-Seq, Pseudo-seq and PSI-seq, which allow transcriptome-wide Ψ mapping [28,52,133]. A variation for A-to-I editing detection, termed Inosine Chemical Erasing-Seq (ICE-Seq), uses cyanoethylation of exiting inosines, which introduces bulky groups allowing to map inosines indirectly at terminated cDNA reads [55]. In addition, RT-mediated errors at specific sites were shown to be caused by RNA modifications rather than the infidelity rate of RTs [134]. Indeed, positional information on m^1^A can be inferred indirectly both from aborted cDNA synthesis and from increased nucleotide misincorporation [135]. In addition, chemical deamination using sodium bisulfite is the basis for indirect m^5^C mapping in RNA using NGS. Methylated cytosines are more refractory to deamination than non-methylated cytosines, which allows determining m^5^C in its sequence context by a method called RNA bisulfite sequencing [82]. Furthermore, differential RNA stability has been exploited to indirectly map specific RNA modifications. For instance, chemical resistance to nucleophilic cleavage under alkaline conditions of phosphodiester bonds located at 3′ of 2′-O-Me residues is the basis for RiboMethSeq, which allows indirect mapping of 2′-O-Me in RNA using NGS [136].

### 4.3. Indirect modification mapping after prior enrichment

Various approaches aim at enrichment of modified RNAs before NGS. The most widely used employ antibodies against nucleic acids and their modifications. Because the majority of covalent RNA modifications involve methyl groups [72,137], various antibodies against methylated nucleosides are used for enrichment of RNA fragments containing methylated nucleotides. In addition, UV-induced cross-linking strategies have been incorporated into antibody-mediated enrichment strategies. Photo-cross-linking-assisted m^6^A sequencing (PA-m^6^A-seq [138]) combines photoactivatable and ribonucleoside-enhanced cross-linking and immunoprecipitation (PAR-CLIP). Alternatively, m^6^A-CLIP and m^6^A individual-nucleotide-resolution cross-linking and immunoprecipitation (m^6^A-iCLIP) use cross-linking-induced mutation and RT truncation profiles to reveal the precise position of m^6^A [25,139]. Furthermore, various mechanism-based enrichment approaches exploit the catalytic steps performed by RNA modification enzymes. For instance, pyrimidine-modifying enzymes can be trapped in denaturant-resistant complexes on substrate molecules containing nucleotide analogues, which has been exploited to enrich for RNA substrates of human Dnmt2/Trdmt1 and NSun2 enzymes using a mechanism-based enrichment method termed Aza-immunoprecipitation, Aza-IP [140]. In addition, mutating particular cysteines in (cytosine-5) RNA methyltransferases allows covalent trapping of substrate RNAs by a method termed methylation-induced cross-linking and immunoprecipitation (miCLiP) [78]. Alternative enrichment strategies employ chemical modification of already modified nucleosides. For instance, selective labelling of Ψ using a chemically synthesized CMC derivative, azido-CMC (N3-CMC), allows conjugating biotin followed by enrichment of biotinylated Ψ-containing RNAs and sequencing by a method called CeU-seq [141]. Also, repurposing the chemical reactivity of known naturally modified nucleosides to create hyper-modifications amenable to selective physical enrichment has been discussed by Phelps *et al*. [142]. The interested reader is referred to recent excellent reviews that detail the involved methodologies [143,144].

## 5. Where might RNA modifications be?

Although major advances have been made in developing NGS-based modification mapping technologies, extensive bioinformatics analysis is needed for extracting experimental noise from potential RNA modification signatures. These algorithms employ user-defined stringencies/thresholds and use diverse statistical methods for ‘modification calling’. Bioinformatic output is often taken for granted and not further addressed using alternative methodologies.

### 5.1. Trust the mapping data?

For instance, RT-mediated ‘modification calling’ relies on significant numbers of reads with specific mis-incorporations or terminations at particular positions. Still, the error rate of Illumina-based sequencing [145] might call artefacts or preclude the accurate detection of less abundant modifications or of modifications that only occur in a small subset of cells or transcripts. Chemical pre-treatment of RNAs might introduce artefacts, as has been observed for RNA bisulfite sequencing [146]. In addition, genomic SNPs might be interpreted as actual RNA modification-induced RT mismatches, and mapping reads across splice junctions or at polyadenylation sites may lead to inaccurately calling false positives, which is a major pitfall for RNA editing mapping, especially in non-model organisms. Considerations of arising problems and suitable mapping tools are reviewed by Conesa *et al*. [147].

### 5.2. Trust the antibodies?

Importantly, RNA enrichment approaches are prone to the creation and inclusion of experimental artefacts. For example, antibody-based enrichment of modified RNA fragments critically depends on sufficient antibody specificity. Antibody specificity, although often taken for granted, is indeed a great concern in medical sciences [148]. Serious problems with insufficient antibody specificity have also been encountered in epigenetics research, which crucially relies on mapping histone modifications. For instance, one of the first and widely used antibodies against methylated H3K9 was later shown to significantly cross-react with H3K27 methylation [149], and the quantitative assessment of antibody specificities used for modification detection showed substantial cross-reactivity between antibody preparations [150,151]. Most natural antibodies against nucleic acids are directed either against double-stranded or ribonucleoprotein structures [152]. However, antibody titres against modifications can be induced by immunization with nucleic acid conjugates containing modifications [153,154]. Importantly, only a small portion of a nucleoside (such as a chemical modification) frequently represents the major antigen epitope surface [155], indicating that antibodies to such restricted epitopes will probably bind to other (structurally related) haptens. For example, anti-m^7^G antibodies cross-reacted with guanosine [155], and electron microscopy revealed that a single anti-m^7^G antibody bound to the m^7^G-cap, while an average of three anti-m^7^G antibodies bound randomly to the remaining RNA molecule [156]. As many covalent RNA modifications involve methyl groups, antibodies against methylated nucleosides could cross-react with similar methyl group-containing epitopes. In addition, some nucleotides contain more than one chemical modification and antibody preparations against one epitope might enrich nucleotides with additional modifications. As an example, anti-m^6^A antibody preparations were unable to distinguish m^6^Am (ribose-methylated m^6^A) from m^6^A in RNA [37].

### 5.3. One method to rule them all?

A much-awaited alternative for single-molecule and single-nucleotide resolution mapping is direct RNA sequencing, which does not require RT. This so-called ‘nanopore’ technology was able to directly detect RNA modifications [157–159]. Direct RNA sequencing would also allow addressing the spatial relationship between different RNA modifications on the same transcript, information that no other available technology can provide, but which will be crucial for proving the suggested ‘epitranscriptomic’ interplay of different RNA modifications. However, even if direct RNA sequencing becomes available, the need and urgency for orthogonal validation of detected RNA modifications, preferentially using physico-chemical methods, will remain the same.

## 6. How many modifications, exactly? Determining stoichiometry will be key

Methods allowing absolute quantification and determining correct stoichiometry of individual RNA modifications are still very much needed because, presently, statements about the actual number of individual RNA modifications are made only in approximation, taking into account transcriptome-wide NGS-derived data and mass spectrometry analysis of species-enriched RNA. Especially, NGS data appear to differ when it comes to reproducibility in modification calling, which is exemplified by the low overlap of Ψ signatures obtained from different carbodiimide-based analyses [160], by discrepancies in m^6^A mapping results using MeRIP-seq and m^6^A-seq [121], and by publications that reach contradictory conclusions about the existence of m^5^C in cRNA of mESCs when using RNA bisulfite sequencing [30,161]. Recently, two methods have been developed that aim to address the stoichiometry of m^6^A. One low-throughput method, named site-specific cleavage and radioactive-labelling followed by ligation-assisted extraction and thin-layer chromatography (SCARLET), quantifies m^6^A at candidate loci [162]. Another method, called m^6^A-level and isoform-characterization sequencing (m^6^A-LAIC-seq), allows measuring m^6^A/A stoichiometry in a transcriptome-wide fashion [163]. Present estimates put the ratio of Ψ/U or m^6^A/A at 0.2–0.6% in cRNA. Estimates for m^1^A/A range from 0.015 to 0.054% in cell lines to 0.16% in tissues [26], for m^5^C/C from 0.025 to 0.1% [164], and to about 0.003% for m^6^Am/A [163]. Such values, which, for instance, translate into an average of three modified positions per 1000 nucleotides per mRNA molecule for m^6^A, raise questions as to whether only a few sequence-identical RNAs are modified at the same position or if they are modified only in a few analysed cells (figure 3). Even the absolute quantification of RNA editing sites is still problematic because editing is probably a cell-specific phenomenon creating different mapping readouts in different experimental systems. Ultimately, high-sensitivity mass spectrometry methods will be the most accurate technology to correctly quantify RNA modifications. However, enrichment of specific RNAs at sufficient purity will still be required. Purification of mitochondrial tRNAs using a sequential set-up of NHS-coupled DNA oligonucleotides has already been achieved by ‘chaplet column chromatography’ followed by mass spectrometry analysis of tRNA modifications [165]. Hybridization-based enrichment has been applied to more RNA species using ‘reciprocal circulating chromatography’, which allows automated and parallel isolation of multiple RNAs from a complex RNA mixture [166]. However, determining and quantifying RNA modification patterns in such purified RNAs using mass spectrometry still suffers from low sensitivity. Even advanced mass-spectrometry approaches that also conserve RNA sequence information (LC/MS on RNA fragments obtained from RNase T1 digest) are lagging behind in sensitivity (by several orders of magnitude) when compared with methods that quantify single nucleosides. Still, the development of mass-spectrometry technology is ongoing and the interested reader is referred to excellent reviews that summarize the current state of the art of these approaches [73,167].

## 7. Start to think post-mapping: time for functional experiments

Despite these advances in the RNA modification field, the toolbox for functional studies is still underdeveloped. Thus, molecular insights into the function of individual RNA modifications are still lagging behind. In addition, identifying the molecular interaction networks of RNA modification components, including their expression regulation and subcellular localization, has not been thoroughly addressed. However, deducing these interactions for every modification in every transcript in different cellular and environmental contexts will be an industrial-scale project involving coordinated research efforts akin to the ‘Encode’ and ‘ModEncode’ projects.

### 7.1. Pick an RNA modification site and … disturb it

With thousands of already mapped RNA modification sites, it is time to address their biological functions, which, surprisingly, only a few studies have attempted. A handful of cRNAs have been studied for the consequences of editing at specific sites [168–172]. Similarly, while it is known that misplacing particular modifications in rRNA affects ribosome function [173], no single site-specific mutagenesis for any covalent cRNA modification that would provide evidence for its function has been reported. In fact, only one report exists, in which two m^6^A sites were changed in Rous sarcoma virus-derived RNA. However, the mutant virus was as infectious as controls, indicating no impact of these m^6^A residues on virus infectivity [174]. Importantly, observations that m^6^A methyltransferases might tolerate degeneracy in the extended consensus sequence indicating compensatory methylation at adjacent sites [175] could not be confirmed when point-mutating various m^6^A sites in yeast *in vivo* [176]. Therefore, rather than perturbing the entire editing/modification machinery using genetic mutations, approaches need to be developed that allow specifically depositing, removing or preventing RNA modifications in specific RNAs. Genome editing by, for instance, using modified CRISPR/Cas9 systems [177] will become a pivotal tool. CRISPR/Cas9 technology has already been used to target epigenetic modulators to DNA [178] or to visualize specific RNAs [179], and could be adapted to modulate RNA modification systems accordingly. Importantly, as virtually nothing is known about the interactions of different RNA modifications that have been mapped to the same RNA sequence (but not necessarily to the same RNA molecule), manipulating RNA modifications in close proximity to each other using genome or transcriptome-editing techniques should make *in vivo* testing of their *cis*-interactions feasible.

### 7.2. Choose your experimental playground

The choice of experimental design and model system will influence not only the detection of RNA modifications, but also how to address their dynamics and biological functions. This is highly relevant in the light of the fact that all known RNA modifications contribute to the fine-tuning rather than to the absolute molecular function/fate/destiny of an RNA. For instance, the use of fast-growing, immortalized cell culture systems, while useful for biochemical approaches, might be ill-suited for the robust detection and manipulation of context-dependent RNA modifications. For instance, A-to-I editing studies in most tissue culture cells require ectopic expression of ADARs [180]. By contrast, cancer cell lines often accumulate advantageous genetic aberrations such as duplications of chromosomal regions that also contain RNA modification enzymes such as, for example, the (cytosine-5) RNA methyltransferase NSun2 [181]. In addition, the origin of cells, the donor genotype and their passage number might all influence cellular behaviour, as evidenced by different potentials of induced pluripotent stem cell lines for self-renewal and *in vitro* differentiation [182]. For instance, it remains unclear how m^6^A impacts embryonic stem cell (ESC) pluripotency. It was reported that m^6^A methyltransferase mutants promote ESC self-renewal by maintaining their ground state [183], but also displayed impaired exit from self-renewal and the naive state [184,185], results that indicated different experimental conditions or cell types. Furthermore, context-dependent functions often remain hidden because of limitations as to how to model life in the laboratory. Thus, finding the correct challenges (i.e. stressors) that create conditions amenable for robust experimentation will be an important experimental design goal in RNA modification research. Also, the use of non-model organisms may provide different insights when addressing the biological function of RNA modification systems [186]. In addition, the observed low stoichiometry of many RNA modifications at specific sites necessitates the development of ‘single cell’ approaches. Observed patterns in the localization of individual RNAs (reviewed in [187]) indicate compartmentalized RNA metabolism and the possibility of localized translation [188], including the possibility of localized RNA modification events. Finally, measuring the actual effects of individual RNA modifications in specific RNA molecules on protein translation is presently the biggest knowledge gap, which cannot be bridged by simply correlating RNA expression data with mapped RNA modification patterns but will require the use of ribosome profiling and stable isotope labelling techniques or the development of additional, preferably single-cell technologies, allowing reproducible quantification of protein translation.

### 7.3. Mechanistic details will be revealed *in vitro*

However, it remains to be seen if *in vivo* manipulation of single RNA modification sites (within a transcriptomic ‘ocean’) will make understanding mechanistic principles easier. Alternatively, further development of simplified *in vitro* reconstitution assays using specifically modified but homogeneous RNA species for functional tests will be useful for identifying mechanistic details such as site selection, protein-binding capabilities as well as structural consequences of particular RNA modifications. Importantly, parameters such as cap-dependency of translation, decapping efficiency, codon usage or splicing in the context of specific mRNA modifications have already been addressed using *in vitro* assays [37,70,189,190], and could be further modified, including real-time kinetic read-outs.

## 8. Conclusion

The explosion in RNA modification-related research during the last decade has mostly been fuelled by the success in identifying RNA modifications in their sequence context. First common conceptual denominators as to why RNA modification systems are so abundant and diverse have been delineated. These include that RNA modification systems facilitate discriminating RNAs from one another and that their activities become necessary when adjusting the functionality of specific RNAs to cellular processes (i.e. translation) that need fine-tuning, especially after environmental perturbation. However, neither transcriptome-wide maps of RNA modifications nor simply disrupting RNA modification systems by mutating writers/readers/erasers, while providing positional information and a diversity of phenotypes, will necessarily lead to a better understanding of the probably complex interplay of RNA modifications, their molecular consequences or their context-dependent regulation. Developing functional assays that incorporate positional information from existing high-throughput data will therefore be the challenge for future generations of RNA biologists.
